# Editorial

**Published:** 1864-07

**Authors:** 


### C. S. Medical & Surgical Journal.

RICHMOND, JULY, 1864.

*AYRES & WADE.....PUBLISHERS AND PROPRIETORS.*

### EDITORIAL.

### *Indigenous Remedies of the South.*

A bountiful Providence has spread over the broad surface of our Southern land, all the elements of an independent nationality. The people trusting in the justice of their cause and the resources which surround them, meet with composure their long isolation from the world, and fall back easily and successfully upon themselves and their own products, for all the requirements of life.

The great drain upon the Medical Department from the large number of sick and wounded necessarily attendant on the great levy, *en masse*, of troops for the defence of the country, early forced itself upon the attention of the Surgeon General. The war opened with the proclamation of the enemy that medical and surgical instruments and appliances were considered contraband of war, and this atrocious step * the forerunner of the many kindred acts of barbarity which have marked the progress of this unholy struggle, compels the department to exert its utmost energies to develope the indigenous medicinal agents of the Confederacy. The accompanying supply table, announced from the Surgeon-General's office as being ready for issue to the medical staff, gives satisfactory evidence of these labors.

A most gratifying progress has also been made in the manufacture of chemicals within our own limits. Blue mass of the best quality, nitrate of silver, sweet spirits of nitre, iodide of potass, and many other leading preparations are prepared on a large scale. Botanical gardens and farms are flourishing at various points. Manufactories and laboratories are rising up in every direction, under the wise supervision of our medical chief; and by consulting the accompanying note the reader will see that even amid the wilds of the Trans-Mississippi, fluid extracts and tinctures of the native remedies of the

*At a recent meeting of the American Medical Association in Chicago, a distinguished member of the profession made a noble effort to wipe away this disgraceful stain from his country's escutcheon. Dr. Gardner, of New York, introduced a preamble and resolutions petitioning the Northern government to repeal their orders to consider medical and surgical appliances contraband of war. This gentleman showed that such cruelties rebounded on their own soldiers, many of whom, in the hands of the rebels, shared the suffering resulting from such a policy, while the act itself was worthy of the dark ages of the world's history.

It will surprise our foreign brethren to know that this learned and powerful tribunal of the medical profession of the North, forgetful of the noble and unselfish teachings of the healing art—blind to all save the gratification of a ruinous hate and ungratified revenge—hissed the benevolent brother from the hall.

country have been successfully used by the surgeons of that distant department.†

We expcet frequently to ask the attention of the reader to this important subject. Nor should we fail here to notice the useful and laborious effort of Surgeon Porcher, in bringing before the public in his work on the Resources of the Southern Fields and Forests the amount of useful material at hand. This valuable essay will receive shortly a careful notice. We will also seize every opportunity of publishing any paper or prescription elucidating the value of the indigenous remedies of the supply table. Our limited space forbids the publication of these reports, *in extenso*, but they will be grouped in the form of abstracts, giving credit to the authors, for their efforts in this very important field. ‡

† Confederate laboratories are in successful operation at the following points:

Trans-Mississippi Department—Surgeons W. R. Johnston and C. D. Curtman, first at Arkadelphia, Arkansas, now at Tyler, Texas. Atlantic Department—Surgeons Piggott, Lincolnton; J. T. Johnson, Charlotte; Chisholm, Columbia; Blackie, Atlanta; Priôleau, Macon; Miller, Mobile; Anderson, Montgomery.

‡ Surgeons Craighill and Lewis and Assistant-Surgeon Bell report, through the Surgeon General's office, some comparative experiments with the indigenous remedies—especially remarking on the combination of the Prodophlyum Peltatum and Juglans Cinerea, in the form of extract, as an aperient and cathartic remedy.

These gentlemen, as well as Surgeon McLaughlin, all of Lynchburg Hospital, make favorable notice of the decoctions and syrups of the Quercus Alba and Rubus Villosus in chronic diarrhœa. The tincture of Cornus Florida and Rubus Villosus making a good astringent tonic, and the decoction Humulus Lupulus and Cucurbita Citrullus as a sedative and tonic draught.

The well known virtues of the Wild Cherry bark and the Polygala Senega are especially mentioned, and the recent observations of Surg. Claiborne, of Petersburg, published in a former number of this journal, as to the value of the Phytolacca Decandra and Rumex Crispus in the treatment of Camp Itch, is amply corroborated by our Lynchburg brethren.

### *Standard Supply Table of the Indigenous Remedies for Field Service and Sick in General Hospital.*

| Botanical Names. | Common Names. | Medical Properties. | Dose. | Form for Issue. |
|---|---|---|---|---|
| Aconus calamus, | Calamus, | Aromatic, stimulant and stomachic, | 10 to 20 grs. | Pulv. |
| " " | " | " " " " | 1 fl. drachm, | Fl. ext. |
| Arum tryphillum, | Wake robin, or Indian turnip, | Expectorant; stim. to gland. system, lungs and skin; in emulsion, | 10 grs. | Pulv. |
| Aristolochia serpentaria, | Virginia snake root, | Stimulant, tonic and diaphoretic; in infusion, | 1 or 2 ozs. | Rad. |
| Asarum canadense, | Wild ginger, | Aromat. stimulant, tonic and diaphoretic, | 20 to 30 grs. | " |
| " " | " " | " " " " " | ½ to 1 fl. drachm, | Fl. ext. |
| Asclepias tuberosa, | Pleurisy root, or butterfly weed, | Diaphoretic; in decoction, | 1 teacupful, | Rad. |
| " " | Do. do. | Expectorant, | 20 to 60 grs. | Pulv. |
| Capsicum, | Pepper, | External irritant, | | Pod, |
| " | " | Stim. stomachic; in gargles, | ½ to 2 drachms, | Tinct. |
| Cassia marilandica, | American senna, | Cathartic; in infusion, | 1 to 3 ounces, | Fol. |
| " " | " " | " " | 1 to 4 drachms, | Fl. ext. |
| Chenopodium Anthelminticum, | Worm seed, | Anthelmintic, in emulsion with ol. ricini, | | Sem. |
| Chimafila umbellata, | Pipsisseway, | Diuretic; in decoction, | 1 pint during 24 hours, | |
| Conium maculatum, | Hemlock, | Narcotic and sedative, | 2 to 3 grs. | Solid ext. |
| Cornus florida, | Dogwood, | Tonic, astringent, | 20 to 60 grs. | Pulv. |
| " " | " | " " in decoction, | 2 fl. ounces, | Cort. |
| " " | " | Tonic, astringent, | 10 to 30 grs. | Solid ext. |
| " " | " | " " | 1 fl. drachm, | Co. fl. ext. |
| Cucurbita citrullus, | Watermelon, | Diuretic, in infusion, | Ad libitum, | Sem. |
| " pepo, | Pumpkin, | Anthelmintic, in emulsion, | 2 ounces, | " |
| Cytisus scoparius, | Scotch broom, | Diuretic, in decoction, | ½ to 1 pt. during 24 hours, | |
| Datura Stramonium, | Jamestown weed, | Narcotic; anti spasmodic and anodyne; tinct. and infusion as local application, | | Fol. |
| " " | " " | Internally (local applic. also for ung. stramonium,) | ¼ to ½ grain, | Solid ext. |
| Diospyros virginiana, | Persimmon, | Tonic; in comp. infusions, and gargles, | | Cort. |
| " " | " | Astringent, | ½ to 1 drachm, | Tinct. |
| " " | " | " | 10 to 30 grs. | Pulv. |
| Erigeron philadelphicum, | Fleabane, | Diuretic; in infusion, | 1 pint during 24 hours, | |
| " canadense, | " | " and astringent; in infusion, | 2 to 4 fl. ozs. | Plant, |
| " " | " | Styptic, | | Oil, |
| Eupatorium perfoliatum, | Boneset, | Tonic, diaphoretic; in infusion, | 2 to 4 fl. ozs. | Herb, |
| Euphorbia ipecacuanha, | Ipecacuanha spurge, | Emetic, | 15 grs. | |
| " corrollata, | Large flowery " | Diaphoretic, | 5 grs. | Rad. |
| Frasera walteri, | American columbo, | Tonic; in infusion, | 1 to 2 fl. ozs. | " |
| Gaultheria procumbeus, | Partridge berry, or spicy wintergreen, | Stim. aromatic, | | Oil, |
| Geranium maculatum, | Cranesbill, | Astringent, in decoction, | 1 to 2 fl. ozs. | Rad. |
| " " | " | " | 10 to 15 grs. | Solid ext. |
| Gentian catesbei, | American gentian, | Tonic, comp. infusion, | 1 to 3 fl. ozs. | Rad. |
| " " | " " | " | 10 to 30 grs. | Solid ext. |
| Gillena trifoliata, or gillena stipulacea, | Indian physic, | Emetic, | 20 to 30 grs. | Pulv. |
| Humulus lupulus, | Hop, | Tonic, hypnotic, in infusion, | 2 fl. ozs. | |
| " " | " | " " " | 1 to 3 drachms, | Tinct. |
| Hyosciamus niger, | Henbane, | Anodyne, soporific, | 1 to 3 grs. | Solid ext. |
| " " | " | " " | 1 fl drachm, | Tinct. |
| Juglans cinerea, | Butternut, | Aperient, cathartic, | 20 to 30 grs. | Solid ext, |
| Juniper communis, | Juniper, | Stim. diuretic, in infusion, | 1 pint during 24 hours, | Berry, |

*Supply Table for Hospitals—Continued.*

| BOTANICAL NAMES. | COMMON NAMES. | MEDICAL PROPERTIES. | DOSE. | FORM FOR ISSUE. |
|---|---|---|---|---|
| Laurus sassafras, | Sassafras, | Stim. aromatic, adjunct to infusions, | | Cort. |
| " " | " | Demulcent, | | Pith, |
| " " | " | Stim. carminative, | 2 to 10 drops, | Oil, |
| Lavandula, | Lavender, | Stim. aromatic, | 30 to 60 drops, | Comp. spts. |
| Leontodon taraxacum, | Dandelion, | Alterative, | 1 fl. drachm, | Fl. ext. |
| Liriodendron tulipifera, | Tulip tree, | Stim. tonic, diaphoretic, | ½ to 2 drachms, | Pulv. |
| " " | " " | " " " | 1 to 3 fl. drachms, | Co. fl. ext. |
| Lobelia Inflata, | Lobelia, | Expectorant, | 1 to 2 fl. drachms, | Tinct. |
| Mentha piperita, | Peppermint, | Arom. stim. and anti-spasmodic | 1 to 3 drops, | Oil, |
| " viridis, | Mint, | " " in infusion, | Ad libitum, | Herb, |
| Monarda punctata, | Horsemint, | Stim. carminative, also adjunct to liniments, internally, | 2 to 3 drops, | Oil, |
| Panax quinquefolium, | Ginseng, | Demulcent, | | Pulv. |
| Papaver, | Poppy, | Anodyne, local application, | | Heads, |
| Phytolacca decandra, | Poke root, | Alterative, for other uses, see Dispensatory, | 1 to 5 grs. | Pulv. |
| Pinceneya pubens, | Georgia bark, | Tonic and antiperiodic, in infusion, | 2 to 3 fl. ozs. | Cort. |
| " " | " " | " " " | 1 drachm, | Pulv. |
| Podophyllum peltatum, | May apple, | Cathartic, | 5 to 15 grs. | Solid ext. |
| Polygala senega, | Seneka snake root, | Stim. and expectorant, in decoction, | 2 fl. ozs. | Rad. |
| " " | " " " | " " " | 1 fl. drachm, | Syrup, |
| Prunus virginiana, | Wild cherry, | Tonic and sedative, in infusion, | 1 to 3 fl. ozs. | Cort. |
| " " | " " | " " " | ½ fl. oz. | Syrup, |
| Quercus alba, | White oak, | Tonic, local application, fomentation, gargle, &c. | | |
| " " | " " | Astringent in decoction, | | Cort. |
| " " | " " | " | ½ to 1 drachm, | Pulv. |
| Rhus glabra, | Sumach, | Astringent, infusion a cooling refrigerant drink in fevers, for gargles, | | Berries, |
| Rubus villosus, or rubus trivialis, | Blackberry, or dewberry | Tonic, astringent; in decoction, | ½ to 2 fl. ozs. | Rad. |
| Do. do. | Do. | " " | 1 fl. drachm, | Comp. syr. |
| Sabbatia angularis, | American centaury | " in infusion, | 2 fl. ozs. | Herb. |
| Salix albra, | White willow, | " astringent; in decoction, | 2 fl. ozs. | Cort. |
| Salvia, | Sage, | " " for gargles, &c., | | Fol. |
| Sanguinaria canadensis, | Puccooon or blood root, | Stim. expectorant, alterative, | ½ fl. drachm, | Tinct. |
| Sarsaparilla, | Sarsaparilla, | Alterative, | 1 fl. drachm, | Fl. ext. |
| Sesamum indicum, | Bene plant, | Demulcent; in infusion, | Ad libitum, | Fol. |
| Solanum dulcamara, | Bitter sweet, or woody nightshade, | Narcotic, alterative; in decoction, | 2 fl. ozs. | Herb. |
| " " | Do. do. | " " | 5 to 10 grs. | Solid ext. |
| Spigelia marilandica, | Pinkroot, | Anthelmintic, | ½ fl. oz. | Co. fl. ext. |
| Spiræa tomentosa, | Hardhack, | Tonic, astringent, | 5 to 15 grs. | Solid ext. |
| Statice caroliniana, | Marsh rosemary, | Astringent; in cold infusion, | | Rad. |
| Stillingia sylvatica, | Queen's root, | Alterative; in decoction, | ½ to 2 fl. ozs. | " |
| " " | " " | " | 1 fl. drachm, | Tinct. |
| Symplocarpus fœtidus, | Skunk cabbage, | Antispasmodic, narcotic, expectorant, | 10 to 20 grs. | Pulv. |
| Triosteum perfoliatum, | Fever root, | Cathartic, | 10 to 20 grs. | Solid ext. |
| Ulmus, | Elm, | Demulcent; in infusion, | Ad libitum, | Cort. |
| " | " | " " | " " | Pulv. |
| Uva ursi, | Bear berry, | Astringent, tonic, with direction to urinary organs: in decoction, | 1 to 2 fl. ozs. | Fol. |
| Veratrum viride, | American Hellebore, | Sedative, expectorant; to be used with caution, | 4 to 8 drops, | N'wood's tinct. |

